# Digitally Enhanced Mentoring for Immigrant Youth Social Capital: Protocol for a Mixed Methods Pilot Study and a Randomized Controlled Trial

**DOI:** 10.2196/16472

**Published:** 2020-03-17

**Authors:** Rebecca Lynn Radlick, Petra Svedberg, Jens M Nygren, Sarah Przedpelska, Deede Gammon

**Affiliations:** 1 Norwegian Research Centre Bergen Norway; 2 School of Health and Welfare Halmstad University Halmstad Sweden; 3 Catalysts Association Oslo Norway; 4 Center for Shared Decision-Making and Collaborative Care Research Oslo University Hospital Oslo Norway; 5 Norwegian Centre for E-health Research University Hospital of North Norway Tromsø Norway

**Keywords:** social capital, e-mentoring, youth mentoring, health promotion intervention, mixed methods, randomized controlled trial, immigrant

## Abstract

**Background:**

There are large disparities between immigrants and native Norwegians in domains such as health, education, and employment. Reducing such disparities is essential for individual and societal well-being. Social capital is associated with positive effects on these domains, and mentoring programs have the potential to boost social capital. However, few studies have assessed mentoring as a social capital intervention among youth or the potential barriers and facilitators of implementing digitally augmented mentoring.

**Objective:**

The goal of this paper is to describe a protocol for assessing the implementation and effectiveness of a digitally augmented mentoring program for immigrant youth as a health intervention to promote social capital. The two-stage analytical framework for a pilot study followed by a randomized controlled trial (RCT) is presented. The pilot aims to assess program fidelity and make necessary intervention adjustments before the RCT. The RCT aims to assess the effects of the implemented intervention program on social capital and the relationship between program fidelity and effects.

**Methods:**

Both the pilot and RCT will use mixed methods with a process evaluation approach used to structure the intervention and a pre-post test survey component to measure social capital and fidelity of program implementation. Interviews will also be used to enrich the quantitative data from the survey.

**Results:**

The pilot study is scheduled to begin in fall 2019. Based on data analyses in spring 2020, potential adjustments will be made to the intervention, with findings used in preparation for the full-scale RCT study.

**Conclusions:**

Digitally enhanced mentoring programs may be a helpful intervention for providing immigrant youth with tools for increasing their social capital and indirectly improving health outcomes. This protocol provides new knowledge about the implementation and evaluation of such programs.

**International Registered Report Identifier (IRRID):**

PRR1-10.2196/16472

## Introduction

### Background and Context

Globally, significant discrepancies exist between immigrant youth and native-born individuals with regards to school dropout, isolation, unemployment, and health. For example, 71% of immigrants in Norway report experiencing at least one of eight listed health problems compared with 49% of the population at large [1]. Relatedly, there is a 26% difference between immigrant and native Norwegian youth with regard to education and employment, with anxiety and depression contributing to these discrepancies [2,3]. These issues also hinder inclusion and integration of new immigrants. Reducing disparities among these vulnerable groups represents an important area of focus, and Norwegian policymakers are increasingly willing to test new approaches.

Social capital, a focus of this study, is associated with a variety of positive health outcomes, including self-reported health [4-6], psychiatric outcomes [7], and mortality [8]. On average, immigrants tend to have less access to certain forms of social capital compared with ethnic Norwegians [9], and investing in social capital can potentially enhance health among immigrant youth. Social capital is multidimensional and has been conceptualized in multiple ways [10-13]. Broadly, it includes relational and cognitive (trust, sense of belonging) and structural (networks or connections among individuals, along with community engagement) dimensions [14]. Analyses can be at the individual or collective level, with focus on different types of social capital: bonding, bridging, and linking [13,15]. With regard to immigrant populations, bonding refers to connection with and support from individuals with a similar ethnic, linguistic, or religious identity, whereas bridging implies connections to those of dissimilar backgrounds, often the majority population [13,16]. Linking relates to vertical relationships between an individual and institutions or individuals in positions of authority [17]. Although bonding social capital is helpful for “getting by,” bridging capital is important for “getting ahead” [13]. Bonding is the most prevalent form of social capital among immigrants [18] and can provide social support and belonging, decrease isolation, and allow sharing of local knowledge [9,19]. Bridging includes benefits, such as increased ability to gather information [20], and can facilitate positive labor market outcomes, such as relevant employment [21]. Both bridging and bonding types are important, and bonding appears to facilitate bridging [22]. Immigrant youth are an important target group for study, as they generally have weaker social capital compared with natives [9,22]. This is exacerbated for the unaccompanied refugees in the group who arrive with no family and lack this important aspect of social capital.

Although approaches for strengthening social capital are clearly worth pursuing, interventions to increase youth social capital, such as the one proposed in this paper, are quite new [23]. Little is known about how such interventions might be implemented, and their effects [24]. Although not systematically studied as such, mentoring programs have the potential to act as “social capital interventions” by assisting immigrants in expanding their networks, and thus their social capital [25,26]. In this setting, mentoring can be defined as “taking place between young persons (ie, mentees) and older or more experienced volunteers (ie, mentors) who are acting in a nonprofessional helping capacity to provide relationship-based support that benefits one or more areas of the mentee’s development” [27]. Participation in youth mentoring programs is associated with improved outcomes across social, behavioral, and academic domains [28]. Such programs, typically conducted by social entrepreneurs, have few traditions in Norway. For the program described in this paper, coordinators recruit, select, train, and match mentors and mentees, and all mentees receive the same basic program. Main program components include training for both mentors and mentees before program start, mentee and mentor sharing of their achievement story (a proud life moment), identification and discussion of mentee strengths, a “roadmap” selecting a goal and describing tasks for the mentee to achieve it, and a network mapping exercise for the mentees. Additionally, the program requires six face-to-face dyad meetings, with one meeting each month during the six-month program period. Staff members also follow up on the matches monthly. Mentees are recently arrived immigrant youth recruited from local schools; willingness and interest in participating are the main selection criteria. Mentor volunteers are recruited in a variety of ways, including personal connections with program staff, social media, and volunteer recruitment websites.

Although one might assume that digital support for this type of mentoring program holds potential for reach and effectiveness, few programs employ digital tools or have been studied systematically [29,30]. Therefore, a prior study [31] of immigrant mentees’ and mentors’ experiences and needs was conducted to guide the development of a digital platform. This platform is integral to the mentoring program described in this protocol. The platform was designed to augment and boost, rather than replace, preexisting mentoring program components. Key elements of the platform include a timeline to provide oversight over dates of program events and show program progress, messaging, cards for identifying personal strengths, a forum for mentees and mentors, a network map, and a toolbox containing helpful supplementary resources (eg, information on writing a good resume). Figures with screenshots illustrating planned iterations of the platform are available in Multimedia Appendices 1 to 4. Although the digital platform is expected to enhance social interactions and program fidelity compared with the program without digital support, the protocol does not directly address this issue.

### Objectives and Significance

This paper describes a protocol for studying a digitally augmented mentoring program as a social capital intervention for immigrant youth in Norway. The protocol proposes a pilot study followed by a randomized controlled trial (RCT). Ultimately, we are interested in assessing the implementation and effects of mentoring program participation on social capital. A process evaluation framework inspired by a previous study [32] will be applied for investigating the implementation of the mentoring program in this pilot study. This framework consists of (1) contextual factors that affect the implementation, (2) what has been implemented and how, and (3) mechanism of impact (ie, the participants’ responses to and interactions with the program). Thus, the objective of the pilot study is to assess program fidelity and design by investigating the context, implementation process, and mechanisms of impact [32,33]. The questions guiding the pilot study are:

1. What contextual factors affect the implementation?
2. How is the mentoring program implemented (ie, fidelity, dose, and reach)?
3. What are the participants’ experiences in the mentoring program?
4. Are the measures used for studying outputs and outcomes valid, and are they acceptable and meaningful to stakeholders?

The pilot allows adjustments of the social capital intervention before full-scale implementation and the RCT. The intention of the RCT is to investigate the effects of the implemented program on the social capital of immigrant youth, guided by the following questions:

1. To what extent was the intervention implemented (outputs)?
2. What are the effects of the implemented program on social capital indicators (outcomes)?
3. What is the relationship between program fidelity and effects?

The study offers both theoretical and empirical contributions. First, much of the work related to mentoring as an intervention has been done in the United States, with primarily qualitative studies in the United Kingdom [28]. Therefore, this work will contribute by extending the empirical context. Second, few studies have assessed social capital interventions [34], particularly among youth. More commonly, social capital is used as an independent variable (see [35] for a health intervention to increase social capital among Latinx and black adults). Furthermore, little is known about the implementation and effects of such interventions [23], although research indicates potential in this area [25,35]. This study seeks to address these gaps, providing both theoretical and summative insights. The overall objective is to evaluate the intervention in light of challenges and opportunities in implementation and to explore the effects of this implementation.

### Analytical Framework

**Figure 1:**
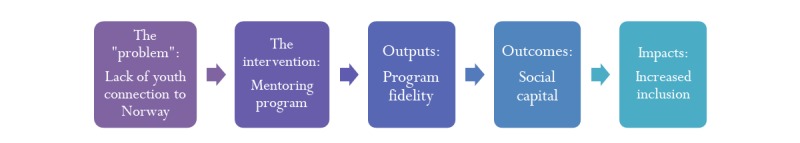
Logic model for the protocol (based on [36,37]).

The logic model [36,37] depicted in Figure 1 provides a valuable framework for identifying and illustrating the relationship between clusters of variables in the study. The model identifies a problem (youths’ lack of connection to Norway), which requires a response, and specifies activities to address the issue (the mentoring intervention). The implementation of the solution leads to tangible outputs (program delivery) and measurable outcomes (social capital) as the consequence of the outputs; the broader impacts (social inclusion) are the logical effects of the intervention. There is a tight relationship between the identified problem, the intervention, the program outputs, and the outcomes of interest.

The pilot study focuses on the first three boxes in Figure 1, allowing adjustments before the RCT if certain outputs are not achieved. The full-scale implementation of the intervention will focus primarily on assessing outputs and outcomes. Impacts are often indirect, and there are significant challenges in causal attribution; thus, impacts are not measured as part of this study. However, the implication is that if outcomes are achieved, broader and more distal societal impacts will result.

The framework depicted in Figure 1 provides a general guide for the study. Research suggests that multicultural youth have narrower networks and less access to certain forms of social capital [9], which contributes to higher dropout rates and poorer health than natives. There is evidence that social capital is associated with improvements in these domains and may facilitate integration and inclusion [4,21].

The mentoring intervention seeks to address these discrepancies. An important focus is on how the program can strengthen mentees’ networks through information exchange and social contact and increase trust and feelings of belonging (social capital). Thus, the study emphasizes outputs and outcomes, as well as the influence of contextual factors on these.

## Methods

### Study Design

The first stage is the pilot study, followed by the RCT. A mixed-methods approach will be used combining quantitative survey instruments (pre-post test design) with qualitative data from interviews and focus groups [38]. The survey will measure outputs (program fidelity, dose, and reach); outcomes (cognitive and structural social capital), which are a result of the implementation outputs; and contextual factors (respondents’ demographics, school characteristics, and usability of the digital platform). Specific cutoffs for assessing fidelity to the core program components (described in the Background and Context section) and effectiveness will be developed based on pilot study results. Interviews with mentors, youth, and program staff will allow a detailed qualitative understanding of the mechanisms occurring and stakeholder experiences.

### Sample and Participant Recruitment

Both stages of the study will be conducted in close cooperation with mentor program staff (the individuals working directly to coordinate, match, train, and follow up on dyads), and with feedback from the youth. The primary unit of analysis for the study is recently arrived immigrant youth, ages 15 to 25 years, attending school. These youth will be in or have completed their first year of Norwegian schooling; therefore, they will have a minimum basic level of Norwegian proficiency. We will offer interviews in Norwegian and English, but will also consider the use of a translator, as necessary, for the RCT to gain a better understanding of the experiences of participants with varying levels of Norwegian language comprehension. Information from mentors and program staff will also be collected. Informed consent will be obtained and the voluntary nature of participation will be emphasized to the youth, with information that they can end their participation at any time. A video will be used to present the research project, handling of data (confidential, but not anonymous), and privacy issues in youth-friendly language to gain fully informed consent and to increase stakeholder (youth) involvement and interest [39].

For the pilot study, all mentees (approximately 40) participating in four of the mentoring programs will be recruited to participate in the survey at the beginning and end of the program. The majority of these mentees have backgrounds from Syria and Eritrea, and almost all will be attending school. At the end of the six-month program, mentors (approximately 40) from these programs will also be invited to respond to a survey. All mentee respondents will receive small denomination gift cards (approximately $28) on completion of the surveys as a gesture of appreciation for their assistance with the project. We will also discuss how the youth perceive receiving gift cards, with a specific focus on any feelings of coerciveness, to make any adjustments before the RCT. Qualitative respondents will be selected from the individuals providing quantitative information to investigate specific aspects of program fidelity and implementation in more detail.

The full-scale RCT will recruit participants from Norwegian schools and include both a control and an intervention group. The study will try to select similar schools for both groups regarding school size, socioeconomic characteristics, and school type (age range of the student body). Within the intervention group of schools, intervention classes will be drawn randomly, whereas control classes will be drawn randomly from the control schools. Participants and nonparticipants will not attend the same schools to avoid contagion effects. The exact number of individuals will be estimated based on a power analysis of data from the pilot study and is anticipated to encompass several hundred youth. Half will be in the intervention group and half in the control group. Students who are not assigned to the control groups for the study will have the opportunity to receive the intervention after the study has concluded. Although all the youth will have a basic level of Norwegian proficiency, additional potential inclusion and exclusion criteria for the RCT will be developed based on findings from the pilot study. Similar to the pilot study, mentors will be surveyed at program conclusion, and respondents will be invited to qualitative interviews and focus groups, to supplement and nuance the quantitative data.

### Data Collection Procedure

#### Pilot Study

After receiving information about the project in the form of a video and giving informed consent, the mentees will receive an individual link to participate in a Web survey. Questions on the mentee survey at program commencement will focus on social capital in the form of relationships with friends, native Norwegians, connectedness to the school environment, and to Norway more broadly. This will be used to test out and adjust measures for the full RCT study based on feedback from the groups. A Web survey will be administered at program completion, with the same survey questions as on the first survey, supplemented with questions about program fidelity (outputs) and a short battery to assess the relationship with the mentor and implementation. Mentors will receive surveys related to program fidelity and the relationship with mentees and staff. Additionally, both mentees and mentors will respond to survey questions on the usability of the digital platform. To supplement the quantitative survey data, qualitative data will be collected from mentors and mentees in interview form for both the pilot and RCT. Individuals will be selected for interviews based on characteristics such as age, sex, immigration background, and length of time in Norway, with a goal to get a very diverse group [38]. Interview questions will address barriers and facilitators to implementation, possible contextual factors relevant to the implementation and participants’ experiences of these factors, and the acceptability and meaningfulness of the measures used in the survey (see Outputs in the Measures section for details on the operationalization of these concepts). Questions to program staff will focus on resources such as program staffing, finances, and technical support for the digital platform.

The pilot study has been approved by the Data Protection Office at Oslo University Hospital. All pilot study data will be stored on a secure remote server as per Oslo University Hospital Personal Data Protection regulations. The pilot study is funded by a grant from the Norwegian Research Council and is a partnership between the Center for Shared Decision Making and Collaborative Care Research at Oslo University Hospital, NORCE-Norwegian Research Centre, Halmstad University, the Norwegian Labor and Welfare Administration (NAV), and Fretex.

#### Full Study With Randomized Controlled Trial

The effects of the program as a social capital intervention will be studied using a two-level clustered randomized trial, with schools randomly assigned as intervention or control schools. The analyses will be at the individual level. In using a randomized comparison of mentees with non-mentees, the effects of mentoring on social capital can be assessed, also controlling for individual differences.

Youth in both the intervention and control groups will complete Web surveys. The youth surveys cover questions related to social capital and will be conducted at baseline (before program commencement), six months after the baseline measurement (at program completion for the intervention groups), and six months after program completion (12 months after the baseline measurement). Surveys to youth in the control group will be administered at the same time points as for the mentees. Mentors will respond to surveys at program conclusion. Because youth in the intervention group will receive mentoring outside of the school environment, we do not have a specific activity planned for the control group. However, control group youth will be put on a waiting list to participate in the mentoring program after study completion, if they desire. Approval from the institutional ethics board (Data Protection Officer) will be applied for and obtained before RCT study commencement.

The following table (Table 1) summarizes the instruments, respondents, and timeline for data collection.

**Table 1:** Overview of the study procedure.

Study stage and data collection instrument		Respondents	Description	Time point
**Pilot**				
	Web survey at program start (baseline)	Mentees (approximately 40)	Social capital: cognitive and structural); demographic variables	Program start (fall 2019)
	Web survey in program middle and conclusion	Mentees (approximately 40); mentors (approximately 40)	Usability of digital platform	Mid and end of program
	Web survey at program completion	Mentees (approximately 40); mentors (approximately 40)	Same as baseline survey; program fidelity	Program completion (6 months after start; spring 2020)
	Interviews	Mentees (5-10); mentors (5); program staff (2)	Acceptability and relevance of social capital measures; barriers and facilitators to implementation and fidelity	Midway and at the end of the program (fall 2019, spring 2020)
**Randomized controlled trial**				
	Web survey at program start (baseline)	Mentees; control group youth	Pre-post social capital measurements; demographic variables	Program start (estimated fall 2020)
	Web survey at program completion	Mentees; mentors; control group youth	Pre-post social capital measurements; program fidelity	Program completion (6 months after program start)
	Postprogram survey after program completion	Mentees; control group youth	Pre-post social capital measurements	Follow-up 6 months after program completion (12 months after program start)
	Interviews	Mentees (10-20); mentors (5-10); program staff (4)	Supplementary information on social capital based on survey responses	Midway and at the end of the program (fall and winter 2020)
**Pilot and randomized controlled trial**				
	Qualitative and metadata from the digital platform	Mentees; mentors	App use data (frequency, length of time, particular modules used); content of forum posts	Throughout the programs

### Measures

Measures to be used for the study survey are adapted from previous research and large-scale cross-national surveys [40-48]. Where relevant, new items were developed specifically for this intervention context, particularly to measure fidelity of implementation.

#### Outputs: Intervention Implementation

Outputs are the process and mechanisms by which the “problem” and its consequences are targeted. More specifically, this is the implementation of the intervention. An important emphasis is on fidelity of implementation of core program components (including dose, or the amount of the intervention, and reach), and mechanisms of impact (participants’ interactions with the intervention) and the contextual factors that have an impact on the implementation (discussed subsequently). Assessment of fidelity is done using a self-developed scale for the study to match the program environment. Program fidelity is measured using the following indicators (previously described under Background and Context): the total number of dyad meetings held with at least one dyad meeting each month (six meetings in all), participation in training before program start, and execution of the main program components, such as network mapping. The proportion of participants that complete the program (reach) will also be assessed. Identification of any deviations or adaptations to these key program components will be noted. Because the intervention also includes a digital component (forums for mentor-mentee contact), log data from the platform, including the number of log-ins and time spent on different parts of the platform, will be analyzed to assess dosage.

Relationship quality (mechanism of impact) will be measured using the 14-item Mentor Strength of Relationship scale [40], adapted from the Big Brothers Big Sisters mentoring context to this context (eg, terminology such as “my Little” is replaced with “my mentee”). Answers are scored on a five-point Likert scale (“strongly disagree” to “strongly agree”). For mentees, the positive statements from the Youth Strength of Relationship Scale [40,41] will be used; response categories are on a five-point scale and range from “not true at all” to “always true.” The Strength of Relationship scales have been assessed for fit using confirmatory factor analysis, with acceptable results [40]. Little research has been conducted on mentee or mentor relationships with program staff and program training. Therefore, self-developed single items will be used that ask to what extent respondents were satisfied with the program coordinator and the training they received (response categories from 1=“very dissatisfied” to 5=“very satisfied”).

#### Outcomes: Social Capital

The outcome variable of interest for the RCT—social capital—includes cognitive and structural dimensions [14]. The cognitive dimension of social capital is operationalized to include youth feelings of belonging, support in relationships, and trust. Feelings of belonging will be measured using a question from the European Social Survey (ESS) Round 8 [42] on how connected the youth feel to Norway. The response scale ranges from 0 (“not emotionally connected at all”) to 10 (“very emotionally connected”).

Youth will also be asked about how often they felt lonely during the previous month (five-point scale: “not at all” to “all the time”) (similar to a question from a Statistics Norway survey on social relations [43] and questions in the ESS [42]).

Shared language is another indicator of cognitive social capital related to belonging [44]. This will be measured by using a self-created question asking: How comfortable are you speaking Norwegian? (scale from 1=“very uncomfortable” to 5=“very comfortable”).

Trust will be measured using the question on generalized trust (A4) from the ESS [42] with a response range from 0 to 10 (“you can’t be too careful” to “most people can be trusted”).

The structural dimension of social capital relates to the presence and patterns of connections between actors or network characteristics (bridging, bonding, linking), school connectedness, and civic engagement or organizational participation. To assess network characteristics, the respondents will be asked about the proportion of their friends with a similar ethnic background and religion (bonding), with an immigrant background (bonding), and with a Norwegian background (bridging). This will be assessed using a five-point scale (“none” to “all”; similar to [22,45]).

Civic engagement will be measured using questions about organizations in which the youth are active. The youth will be provided with a list of different organization types (religious, sports, art and music, volunteer organization) and will be asked to respond if they are a member, have participated previously, or are not a member (similar measures are used in ESS and in the Ungdata survey for Norwegian adolescents [46]).

Patterns and nature of contact with friends and family will be measured in several ways. The youth will be asked if they have at least one good friend that they can fully trust (four-point scale from “no, I have no one I would call a friend at the moment” to “yes, for sure”) (questions are taken from Ungdata [46]). Respondents will also be asked about frequency of contact with their friends outside of school or work [42], with responses on a six-point scale from “never” to “every day” (similar to [45]).

To measure school social capital and connectedness, several dimensions (teachers, classmates, school) will be used. Connectedness will be measured using indicators from the Health Behavior in School-aged Children study protocol questionnaire [47], as used in a Swedish study on social capital [4]. Connectedness with teachers will be measured with three items (five-point scale from “strongly disagree” to “strongly agree”); for example, “I feel that my teachers accept me as I am.” Connectedness with classmates will be measured using three items (eg, “most of the students in my classes are kind and helpful”) with the same five-point scale. These two scales have been validated using confirmatory factor analysis [47]. To assess connectedness with school, three questions from Ungdata [46] (“I am often bored at school,” “I don’t like going to school,” and “It’s important to do well at school”) will be used, also with a five-point scale. One question from the Health Behavior in School-aged Children [47] (“How do you feel about school at present”) will also be used (4-point response scale from “I like it a lot” to “I don’t like it at all”). To assess school attachment, a self-developed item similar to that used in Ungdata [46] will be used to assess if and how often the youth have considered dropping out of school in the previous three months. Response alternatives (four-point scale) range from “never” to “very often.”

#### Context and Controls

Demographic characteristics of the youth in the sample, such as age, sex, length of time living in Norway, and economic status (inquiring about their finances during the past year), will also be included in the survey. Characteristics related to setting (school size and centrality) and the digital context in which the respondents participate will also be taken into account. In the pilot study, the usability of the digital platform will be assessed in a survey, which will include questions from the System Usability Scale [48], a robust tool for analyzing usability [49]. The scale has 10 items (eg, “I thought the platform was easy to use”) and response choices on a five-point scale from “strongly disagree” to “strongly agree.” Assessment of contextual factors will also be collected from mentors and youth in interview form.

### Data Analysis

Quantitative data will be analyzed using statistical software packages. Descriptive statistics will be presented and pre-post measures used to determine the impact of the intervention on social capital and more distal outcomes. Additionally, because the literature suggests that mentoring produces better outcomes for some groups (eg, high-risk male youth) over others [50], we will conduct exploratory analyses to investigate differing effects of the intervention based on age, gender, and time in Norway. All interviews will be recorded and transcribed, and qualitative analysis software (NVIVO 12) will be used on the transcribed interviews and forum data. This will entail coding of responses, first extracting broad themes from the data, and then identifying subthemes [51]. This type of qualitative analysis will supplement the data from the surveys, allowing a better understanding of the implementation process and explain why specific outcomes might have occurred [38].

## Results

This protocol was informed by a prepilot study conducted from January to June 2019 [31], which focused on the experiences and needs of stakeholders with relation to the digital mentoring platform and perceptions around the concept of social capital. This enabled the identification of areas of focus and strategy for this protocol, particularly in relation to social capital and key components of the mentoring program. Therefore, the resulting protocol is considered well-suited for providing a valid analysis of program fidelity (which the mentoring program is currently working to adhere to) and new knowledge about social capital as a health promotion intervention among immigrant youth.

The pilot study will commence in fall 2019 and conclude in spring 2020. The participants (mentors and youth mentees; approximately 40 of each) have already been identified for the pilot portion of the protocol. Efforts related to recruitment for the RCT will begin after the pilot study is finished.

## Discussion

The overall objective of this protocol was to present a plan for evaluating the implementation and effects of digitally augmented mentoring relative to social capital among immigrant youth. The main working hypothesis is that students who receive the intervention will have broader networks and higher levels of trust and feelings of belonging (greater social capital) compared with those students who do not receive the intervention. Considering the paucity of research on social capital interventions, the protocol should be relevant for researchers interested in community-based health promotion and in social capital more broadly. More specifically, it provides a framework for analyzing mentoring programs for immigrant youth to see what works under what circumstances and for whom. However, there are some anticipated limitations.

Because mentees self-select to the program in the pilot study, these findings may not be generalizable to a larger group; however, this is not the primary objective of the pilot. The RCT should ameliorate this issue owing to the random selection of individuals and classes. Another potential limitation relates to language issues, particularly in the survey instrument. The pilot study is intended to assess the relevance and acceptability of the instruments to the target groups; comprehension will also be a relevant consideration at this stage. Thus, this issue will hopefully be minimal in the RCT. Attrition, particularly after program completion, is another potential limitation, as in most survey research.

If this intervention is successful, it may have an impact on the possibility for young people to be better included in Norwegian society. Further work could potentially encompass cost-benefit analyses if there is support for the hypotheses. These preliminary results from the pilot and RCT, if positive, could be promising for a potential expansion of the intervention to other contexts and target groups.

## Appendix

Multimedia Appendix 1 Platform screenshot: Log-in screen with code access.

Multimedia Appendix 2 Platform screenshot: Overview of program activities including program "launch" and "achievement story".

Multimedia Appendix 3 Platform screenshot: Overview of program activities including "roadmap", "network mapping", and "reflection exercise".

Multimedia Appendix 4 Platform screenshot: Information on "network building".

Multimedia Appendix 5 Peer reviewer report from the Norwegian Research Council.

## Abbreviations

ESS: European Social Survey
RCT: randomized controlled trial
