# Supplementary figures and images for: Digitally Enhanced Mentoring for Immigrant Youth Social Capital: Protocol for a Mixed Methods Pilot Study and a Randomized Controlled Trial
Source: JMIR Res Protoc. 2020 Mar 17;9(3):e16472. doi: 10.2196/16472 (PMC7109612; doi:10.2196/16472)

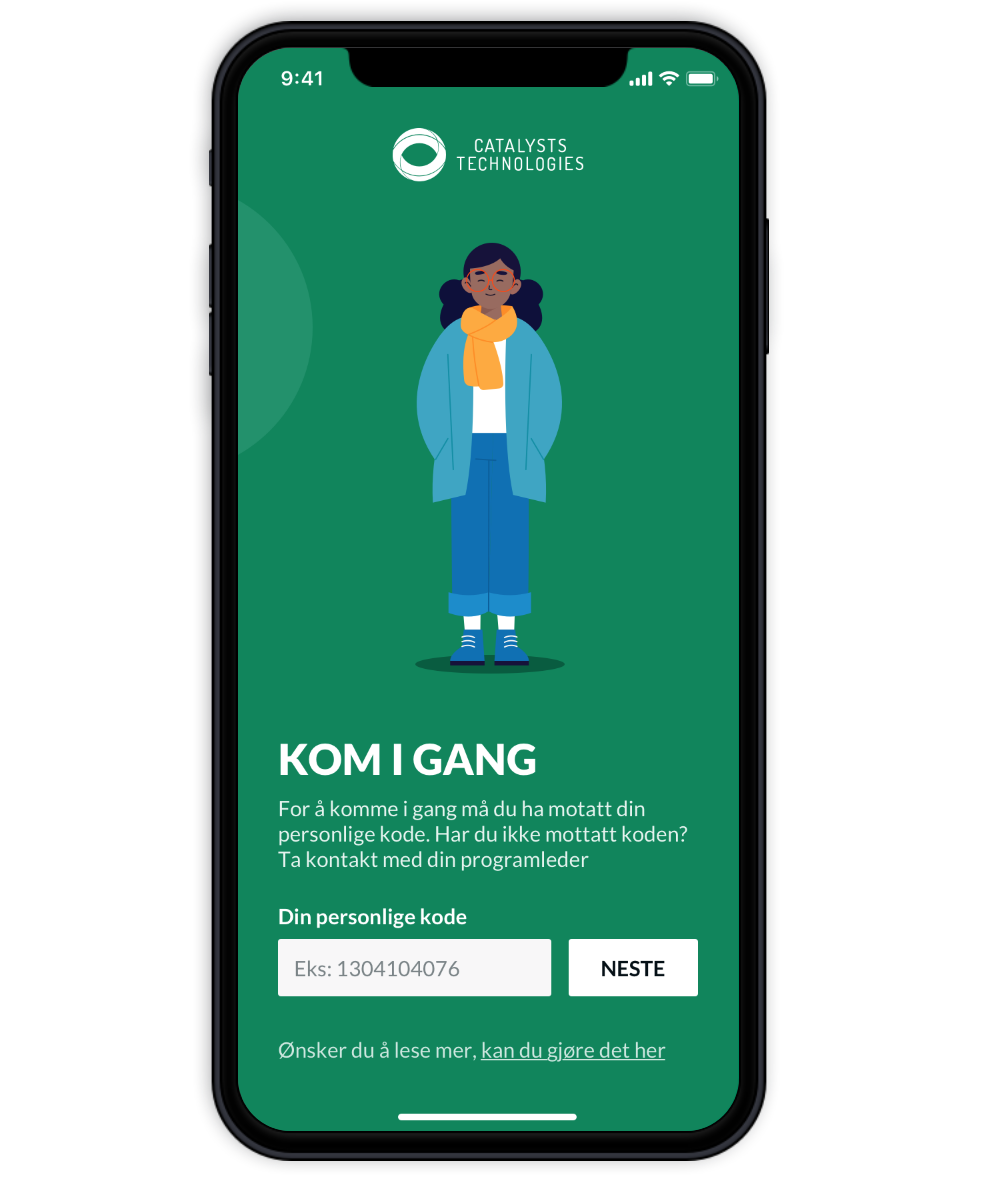

Supplement: Multimedia Appendix 1 [file resprot_v9i3e16472_app1.png]

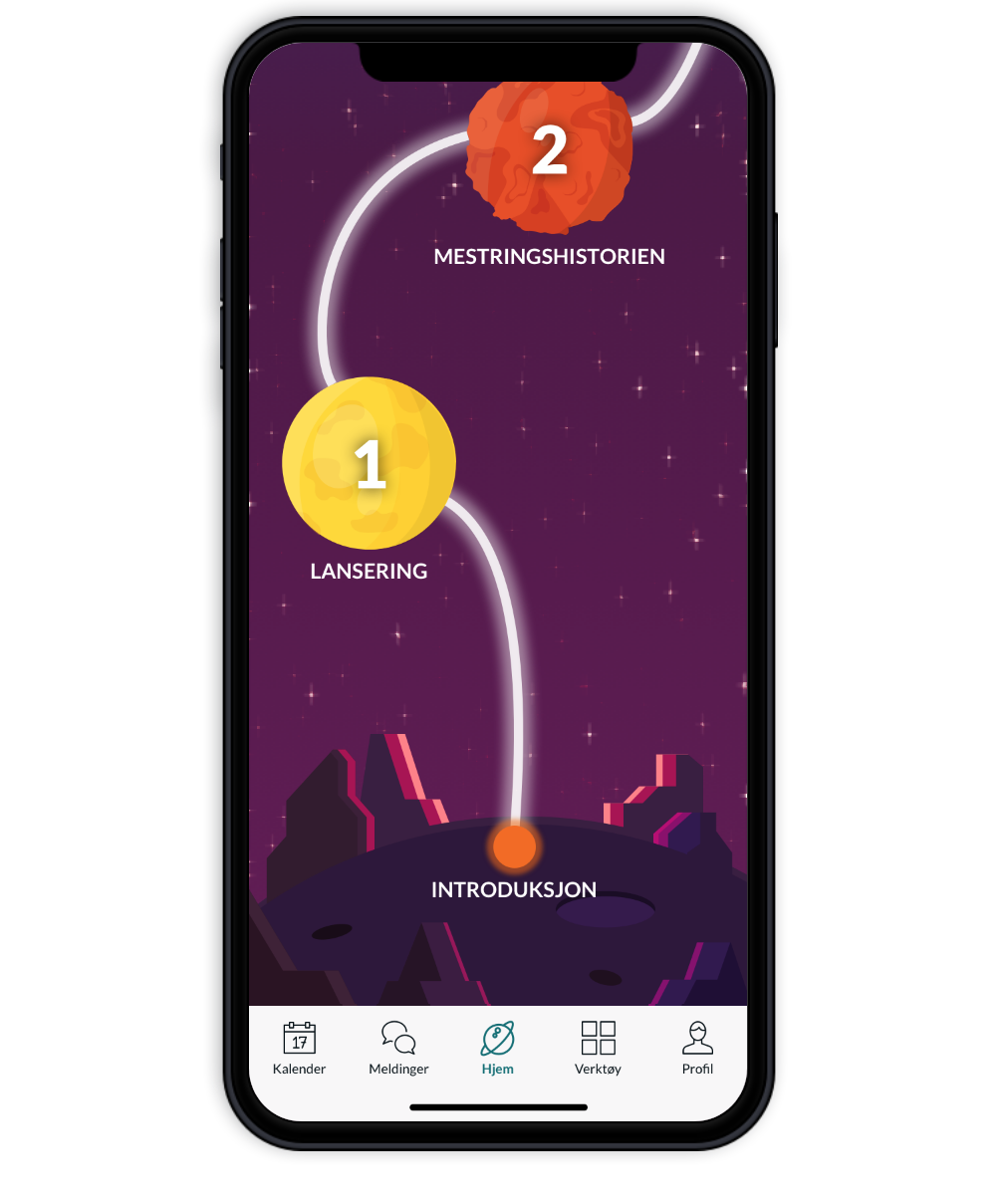

Supplement: Multimedia Appendix 2 [file resprot_v9i3e16472_app2.png]

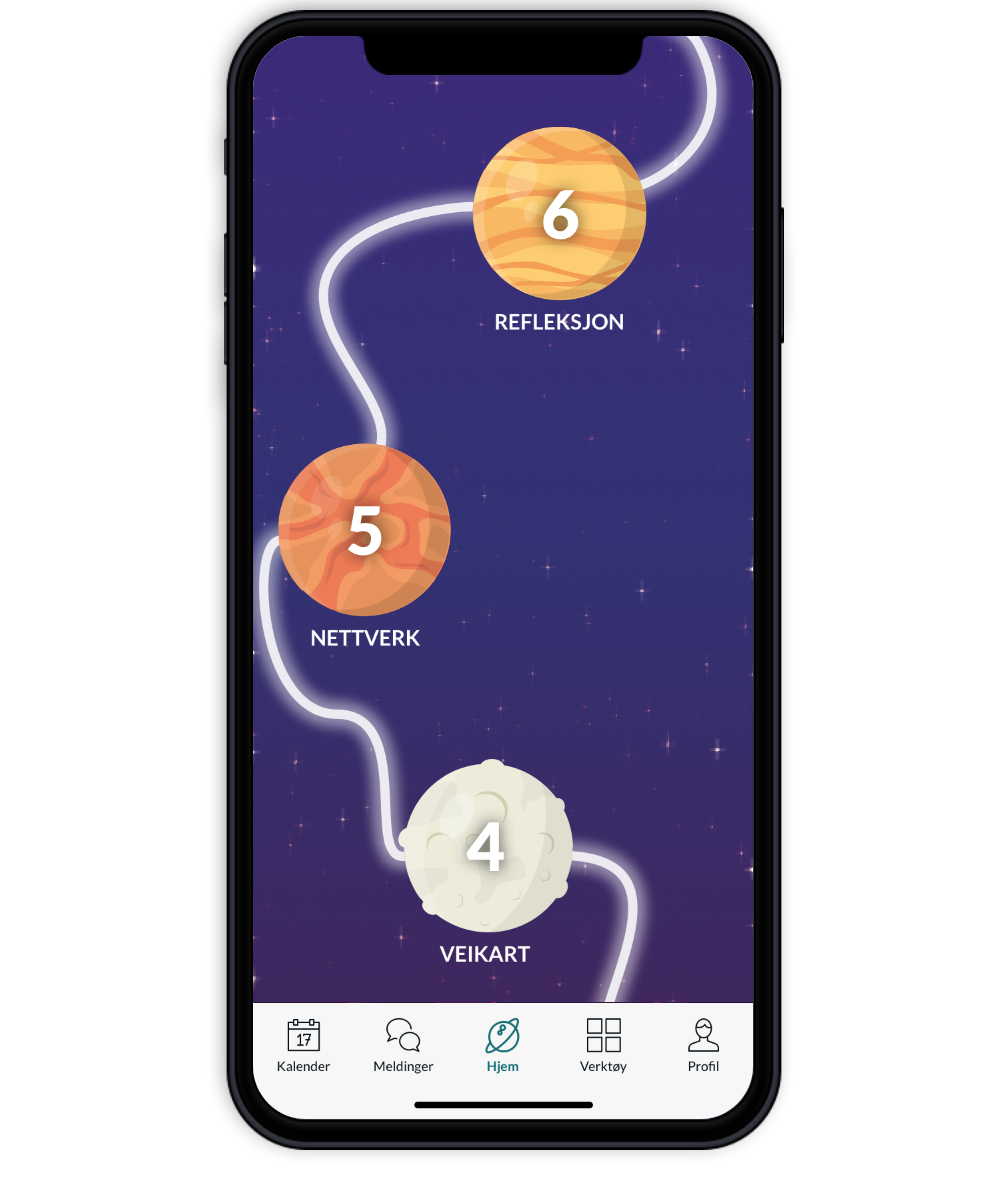

Supplement: Multimedia Appendix 3 [file resprot_v9i3e16472_app3.png]

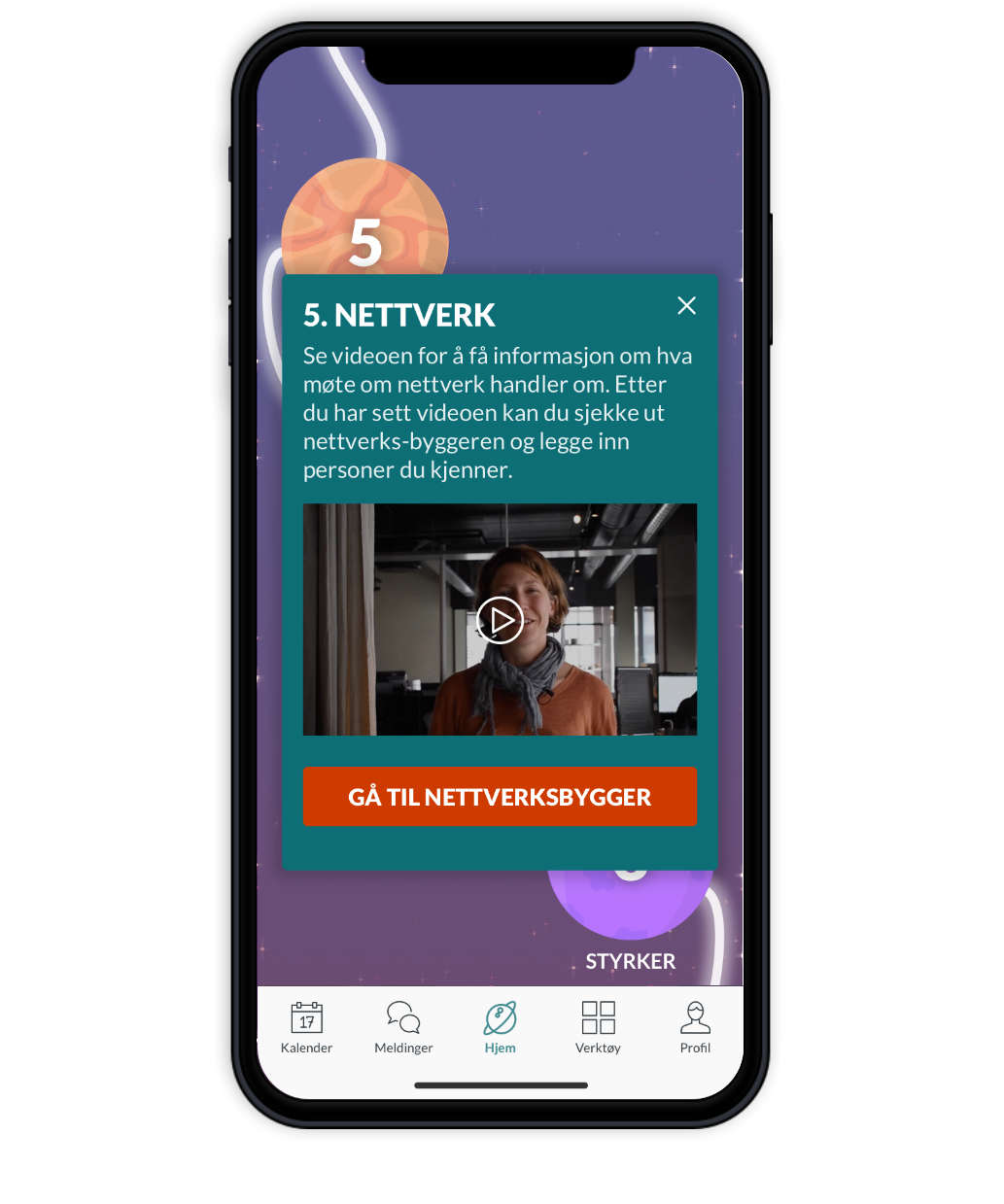

Supplement: Multimedia Appendix 4 [file resprot_v9i3e16472_app4.png]
