# Supplementary material for: Digitally Enhanced Mentoring for Immigrant Youth Social Capital: Protocol for a Mixed Methods Pilot Study and a Randomized Controlled Trial
Source: JMIR Res Protoc. 2020 Mar 17;9(3):e16472. doi: 10.2196/16472 (PMC7109612; doi:10.2196/16472)
Supplement: Multimedia Appendix 5 [file resprot_v9i3e16472_app5.pdf]

Prosjektvurdering - Karaktersammendrag

Prosjektnummer: 269438
Prosjekttittel: Resiliency at Work (R@W) by and for at-risk young people: Digital innovations that promote social inclusion, health and employment

	Vurderingskriterium	Karakter
1	Innovasjonsgrad	5
2	Verdiskapingspotensial for offentlig sektor	5
3	Realisering av innovasjonen i offentlig sektor	4
4	Forskningsgrad	5
5	Prosjektkvalitet for FoU-prosjektet	5
6	Gjennomføringsevne	6
7	Forskningens innovasjonsrelevans	B
8	Addisjonalitet	B
9	Dokumentkvalitet	B
	Hovedkarakter*	5

Karakterskala: 7, 6, 5, 4, 3, 2, 1 (7 er best)
A, B, C (A er best)

*) Hovedkarakter er et uttrykk for hvor godt prosjektet oppfyller intensjoner og formål for søknadstypen med utgangspunkt i bedømmelsen av de ulike kriteriene. Hovedkarakter 3 eller lavere forteller at prosjektet har en avgjørende svakhet i form av karakter 3, 2, 1 eller C på minst ett av kriteriene.

Karakterene er en del av underlaget ved det besluttende organs behandling av søknaden.

Forskningsrådet

Vurdering av søknad sendt til Norges forskningsråd

Søknad

Prosjektnummer	269438
Prosjekttittel	Resiliency at Work (R@W) by and for at-risk young people: Digital innovations that promote social inclusion, health and employment
Prosjektleder	Gammon, Deede
Prosjektansvarlig	Oslo universitetssykehus HF
Program/Aktivitet	Gode og effektive helse-, omso
Saksbehandler	Vidar Sørhus

Bekreftelse

Ved å levere dette skjema i utfylt stand, bekrefter jeg / vi følgende forhold (dette gjelder for den enkelte ekspert eller panelet):

- Jeg/vi er habil til å vurdere denne søknaden. Se Forskningsrådets bestemmelser om habilitet og tillit.	Ja
- Jeg/vi har lest og gjort meg/oss kjent med de kriteriene jeg/vi er bedt om å vurdere søknaden etter og retningslinjene for bruk av karakterskalaen. Karakterskalaen skal brukes for å reflektere absolutte verdier, og karakterer skal ikke settes relativt til andre søknader som panelet/fageksperten vurderer.	Ja
- Jeg/vi er kjent med og har akseptert bestemmelsene for vurdering av søknader for Norges Forskningsråd. Se Bestemmelser for eksperter / panel som skal vurdere søknader for Norges forskningsråd.	Ja
- Jeg/vi er kompetent til å foreta denne vurderingen.	Ja

Karakteroversikt

Kriterier	Karakter
Innovasjonsgrad | IPO	5
Verdiskapingspotensial for offentlig sektor | IPO	5
Realisering av innovasjonen i offentlig sektor | IPO	4
Forskningsgrad | IPN	5
Prosjektkvalitet for FoU-prosjektet | IPN	5
Gjennomføringsevne | IPN	6

Sjekkpunkter	Svar
Brukermedvirkning	Meget godt

Kriterier

Innovasjonsgrad | IPO

Hvor stor er innovasjonsgraden?

Innovasjonsgrad er et uttrykk for hvor betydelig innovasjonen er i forhold til “state of the art” på et område. Det vurderes hvorvidt det er en innovasjon bare for partnerne i prosjektet eller også i nasjonal og/eller internasjonal sammenheng.

Innovasjonsområder:
* Nye eller forbedrede produkter/tjenester
* Nye eller forbedrede metoder for produksjon/leveranse/distribusjon av produkter/tjenester
* Nye eller forbedrede former for ledelse/organisering/arbeidsforhold/kompetanse
* Nye eller forbedrede forvaltnings- og forretningsmodeller

Innovasjonspotensialet er beskrevet og kommer fram i beskrivelsen. Det savnes imidlertid en klarere spesifisering av innholdet i innovasjonen, og derigjennom klargjøring av hva prosjektet vil bidra med som representerer noe kvalitativt nytt.

Valgt karakter: 5 - Meget godt
Tydelig innovasjon i forhold til "state of the art" for området.

Verdiskapingspotensial for offentlig sektor | IPO

Hvor stort verdiskapingspotensial har prosjektet for offentlig sektor og deres brukere?

Verdiskapingspotensial for offentlig sektor er et uttrykk for forventet verdiskaping for offentlig sektor og deres brukere når innovasjonen er realisert. Verdiskapingen skal vurderes opp mot de samlede kostnadene for FoU-prosjektet og realiseringen.

Verdiskapingspotensialet kan fremkomme som:
* Økt effektivitet
* Bedre kvalitet
* Reduserte kostnader
* Økt nytteverdi
* Bedre tjenester
* Bedre verktøy/metoder for beslutningsprosesser
* Andre typer bidrag til verdiskaping

Det vurderes som en styrke at prosjektet legger opp til å koble flere aktører, noe som muliggjør synergieffekter, hvilket vil kunne ha positiv effekt for offentlig sektor.

Valgt karakter: 5 - Meget godt

Prosjektet har et verdiskapingspotensial for offentlig sektor og deres brukere, over det som er normale forventninger på sammenlignbare områder.

Realisering av innovasjonen i offentlig sektor | IPO

Hvor gode er forutsetningene for at verdiskapingspotensialet kan bli realisert?

Realisering av innovasjonen er et uttrykk for i hvilken grad forutsetningene er til stede for at verdiskapingspotensialet kan bli realisert. Det skal forutsettes at FoU-prosjektet blir vellykket.

Disse punktene vurderes:

Plan for realisering av innovasjonen
* Tiltaksplan
* Milepælplan
* Ressursbehov
* Forankring

Risikovurdering og risikohåndtering
* Iverksettingsrisiko
* Finansiell risiko
* Organisatorisk risiko
* Risiko knyttet til beslutningsprosess
* Annen risiko

Forutsetningene for realisering av potensialet er tilstede. Planen er oversiktelig og det er satt opp milepæler. Samtidig er vurderingene av risikofaktorene vel knapp. Herunder hvordan og i hvilken grad offentlig sektor vil ta innovasjonen i bruk.

Valgt karakter: 4 - Godt
Planen har noen svakheter, men det er likevel sannsynlig at deler av verdiskapingspotensialet kan realiseres.

Forskningsgrad | IPN

Hvor høy er forskningsgraden i prosjektet?

Forskningsgrad er et uttrykk for i hvilken grad prosjektet frembringer ny kunnskap av betydning for den faglige utviklingen innen de feltene som forskningen omfatter. Det må fremgå at prosjektet har kunnskap om forskningsfronten.

Forskningsgraden vurderes i forhold til disse punktene:
* Forskning i internasjonal forskningsfront
* Forskningens andel av FoU-prosjektet

Det er lagt opp til omfattende forskning i prosjektet. En innvending er at innholdet i forskningen ikke er tilstrekkelig beskrevet.

Valgt karakter: 5 - Meget godt
Prosjektet arbeider i kontakt med forskningsfronten, og forskningen utgjør en vesentlig andel av prosjektet.

Prosjektkvalitet for FoU-prosjektet | IPN

Hvor god er prosjektkvaliteten for FoU-prosjektet?

Prosjektkvalitet for FoU-prosjektet er et uttrykk for i hvilken grad prosjektet fremstår som gjennomførbart ut fra disse punktene:
* FoU-metode
* Prosjektgjennomføringsplan, inklusiv milepæler og leveranser
* Budsjett og ressursinnsats, herunder finansiering

Prosjektet framstår gjennomførbart med de rammer og organisering som er beskrevet i skissen. Metoden er omtalt, men den kunne med fordel vært problematisert noe mer inngående enn det som er tilfelle.

Valgt karakter: 5 - Meget godt
Både FoU-metode, prosjektplan og ressursinnsats er godt beskrevet og godt tilpasset oppgaven, men med noen svakheter.

Gjennomføringsevne | IPN

Hvor god er gjennomføringsevnen?

Gjennomføringsevne er et uttrykk for i hvilken grad samarbeidspartnerne samlet sett anses å kunne gjennomføre FoU-prosjektet på en best mulig måte.

Følgende vurderes:
* Faglig ledelse
* Organisering og administrativ ledelse
* Samarbeidskonstellasjonen (sammensetning, rollefordeling og kompetanse)

Realisering av innovasjonen vurderes ikke.

Gjennomføringsevnen antas å være stor, med erfarne aktører og en sterk faglig leder.

Valgt karakter: 6 - Svært godt
FoU-prosjektet har samarbeidspartnere med gode faglige og ledelsesmessige kvalifikasjoner. Samarbeidskonstellasjonen og prosjektorganiseringen gir svært gode forutsetninger for at prosjektet i hovedsdak kan nå sine mål.

Sjekkpunkter

Sjekkpunkter	Svar
Brukermedvirkning	Meget godt

Kommentar til sjekkpunkter

Flere aktører med erfaring og det er lagt opp til tett samarbeid.
